# Multidrug Resistance-Associated Protein 2 Expression Is Upregulated by Adenosine 5’-Triphosphate in Colorectal Cancer Cells and Enhances Their Survival to Chemotherapeutic Drugs

**DOI:** 10.1371/journal.pone.0136080

**Published:** 2015-08-21

**Authors:** Valérie Vinette, Morgane Placet, Guillaume Arguin, Fernand-Pierre Gendron

**Affiliations:** Department of Anatomy and Cell Biology, Faculté de Médecine et des Sciences de la Santé, Pavillon de Recherche Appliquée sur le Cancer, Université de Sherbrooke, Sherbrooke, Quebec, Canada; University of Cambridge, UNITED KINGDOM

## Abstract

Extracellular adenosine 5’-triphosphate (ATP) is a signaling molecule that induces a plethora of effects ranging from the regulation of cell proliferation to modulation of cancerous cell behavior. In colorectal cancer, ATP was reported to stimulate epithelial cell proliferation and possibly promote resistance to anti-cancer treatments. However, the exact role of this danger-signaling molecule on cancerous intestinal epithelial cells (IECs) in response to chemotherapeutic agents remains unknown. To address how ATP may influence the response of cancerous IECs to chemotherapeutic agents, we used Caco-2 cells, which display enterocyte-like features, to determine the effect of ATP on the expression of multidrug resistance-associated protein 2 (MRP2). Gene and protein expression were determined by quantitative real-time PCR (qRT-PCR) and Western blotting. Resistance to etoposide, cisplatin and doxorubicin was determined by MTT assays in response to ATP stimulation of Caco-2 cells and in cells for which MRP2 expression was down-regulated by shRNA. ATP increased the expression of MRP2 at both the mRNA and protein levels. MRP2 expression involved an ATP-dependent stimulation of the MEK/ERK signaling pathway that was associated with an increase in relative resistance of Caco-2 cells to etoposide. Abolition of MRP2 expression using shRNA significantly reduced the protective effect of MRP2 toward etoposide as well as to cisplatin and doxorubicin. This study describes the mechanism by which ATP may contribute to the chemoresistance of cancerous IECs in colorectal cancer. Given the heterogeneity of colorectal adenocarcinoma responses to anti-cancer drugs, these findings call for further study to understand the role of P2 receptors in cancer drug therapy and to develop novel therapies aimed at regulating P2 receptor activity.

## Introduction

Colorectal cancer (CRC) involves the abnormal proliferation of intestinal epithelial cells (IECs) resulting from spontaneous genetic alterations or as the result of continuous insults as observed in patients with long-term chronic inflammatory bowel disease [1,2]. Progression from a simple neoplastic lesion to adenocarcinoma involves not only intrinsic factors, such as the expression of oncogenes like *c-MYC* or the repression/mutation of suppressor genes such as *TP53*, *but also* the participation of an array of soluble regulatory factors including cytokines, of which TGF-β is a well-documented pro-tumorigenic factor [1,3]. Recently, extracellular adenosine 5’-triphosphate (ATP) has been identified as a danger-signaling molecule secreted during inflammation and in the tumor microenvironment to attract immune cells and coordinate cancer cells behavior [4,5]. In the tumor vicinity, it was reported that the ATP concentration could reach 100 mM, which is well beyond the concentration required to activate nucleotide receptors [6,7].

Extracellular ATP is the endogenous agonist of the P2X receptor family of ligand-gated ion channels and a limited number of the P2Y subfamily of G protein-coupled receptors, namely the human P2Y_2_ and P2Y_11_ receptors [8]. In solid tumors, such as CRC, ATP was shown to reduce the growth of high-grade bladder cancer cells both *in vitro* and *in vivo* [9]. In clinical settings, ATP infusions to patients with advanced non-small cell lung cancer were found to enhance significantly the quality of life and overall survival in those receiving infusions *vs*. the placebo cohort [10–12]. However, the impact of ATP on intestinal epithelial cancer cells is not as clearly defined. *In vitro*, ATP was reported to increase Caco-2 cell proliferation through the activation of the MAPK signaling cascade [13], leading the authors to suggest that ATP could act as a mitogen on cancerous IECs and potentially be involved in resistance to treatment. Another study reported that high ATP concentrations (> 1 mM) suppressed Caco-2 cell proliferation [14]. It was even proposed that the anti-tumor immunity resulting from chemotherapy may be mediated by ATP release from tumor cells and the activation of the NOD-like receptor family, pyrin domain containing 3 (NLRP3) inflammasome [15], thus suggesting the participation of the purinergic receptor P2X7 [16,17]. However, P2X7 along with P2Y receptors have also been associated with tumor promotion [18,19]. There is thus a clear need to clarify the action of ATP in CRC.

For over 40 years, the mainstream therapy for advanced CRC as been the combination of DNA-damaging drugs such as etoposide or 5-fluorouracil (5-FU) in combination with alkylating agents cisplatin or oxaliplatin as well as doxorubicin and its derivatives [20–24]. Although most cases of CRC are etoposide resistant, there are studies that suggest using this topoisomerase II inhibitor in combination with resveratrol or FTY720 (fingolimod) to avoid drug resistance [24,25]. Nevertheless, a particular class of proteins belonging to the ATP-Binding Cassette (ABC) superfamily may interfere with the efficacy of such treatments due to their ability to export chemotherapeutic agents out of cells [26]. The ABC transporters encompass seven subfamilies (A-G). Family C (ABCC) is composed of 13 members of whom nine have been described as multidrug resistance-associated proteins (MRPs) which include MRP2 (ABCC2) [26]. In cancer, the positive expression of MRP2 has been associated with gallbladder carcinoma aggressiveness and poor prognosis [27] as well as chemoresistance and poor prognosis in patients with esophageal squamous cell carcinoma [28]. Although an increase in MRP2 transcript expression has been measured in colon cancer tissues, no correlation has been made to date between MRP2 expression levels and disease severity or prognosis [26]. Nonetheless, the expression of MRP2 has been significantly associated with increased resistance to cisplatin but not to 5-FU, suggesting a role in colon cancer resistance to selected chemotherapeutic drugs [26,29]. Given the above, the purpose of this study was to investigate whether the stimulation of intestinal epithelial cancerous cells by ATP leads to a modulation of MRP2 expression, which we postulate would entail an increased resistance of tumor cells to certain chemotherapeutic drugs and hence be detrimental to the treatment of colorectal cancer by enhancing the survival of cancer cells.

## Materials and Methods

### Reagents

Dulbecco's modified Eagle medium (DMEM), penicillin-streptomycin, HEPES and fetal bovine serum (FBS) were purchased from Wisent (St. Bruno, QC, Canada). GlutaMax was from Life Technologies (Burlington, ON, Canada). ATP, suramin and pyridoxalphosphate-6-azophenyl-2',4'-disulphonic acid (PPADS) were from Sigma-Aldrich (Oakville, ON, Canada). The MEK1/2 (UO126), p38 MAPK (SB203580), PI3K (LY294002) and NF- κB (BAY-11-7082) inhibitors as well as 3-(4,5-dimethyl-2-thiazolyl)-2,5-diphenyl-2H-tetrazolium bromide (MTT) were acquired from Calbiochem (Mississauga, ON, Canada). Dimethyl sulfoxide (DMSO) was from Fisher Scientific (Ottawa, ON, Canada). The rabbit polyclonal antibodies against MRP2 and β-tubulin were purchased from Cell Signaling Technology (Pickering, ON, Canada). Horseradish peroxidase (HRP)-conjugated donkey anti-rabbit IgG was from Santa Cruz (Santa Cruz, CA, USA) and the ECL reagent from Millipore (Toronto, ON, Canada). The cytotoxic drugs etoposide, cisplatin and doxorubicin were purchased from the chemotherapy pharmacy at the Université de Sherbrooke Hospital Center (Sherbrooke, QC, Canada).

### Cell Culture

The human colon carcinoma cell line Caco-2 (ATCC, HTB37) and human embryonic kidney cell line HEK293T (ATCC, CRL-11268) were grown as previously described [30]. Specific kinase inhibitors were added to serum-free medium 30 minutes prior to nucleotide stimulation as presented in figures. For drug cytotoxicity assays, Caco-2 cells were grown in DMEM medium without phenol red.

### Generation of MRP2 shRNA cell lines

The 21mer shRNA constructs directed against human MRP2 (NM_000392) were purchased from Sigma-Aldrich MISSION shRNA (St. Louis, MO). Lentiviruses were produced in HEK293T cells and used for Caco-2 cell infection as previously described [31]. To validate shRNA efficiency, Caco-2 cells were harvested, and MRP2 expression analyzed by Western blotting and quantitative real-time PCR (qRT-PCR).

### Quantitative real-time PCR

Caco-2 cells were stimulated with 100 μM ATP for 3 and 6 hours. Total RNA was isolated from Caco-2 cells with TRIzol Reagent (Life Technologies) according to the manufacturer’s instructions. Complementary DNA (cDNA) was synthesized from 2 μg of purified RNA by reverse transcription using the SuperScript II system (Invitrogen Life Technologies, Burlington, ON, Canada). Five percent of the synthesized cDNA was used as a template for qRT-PCR using the Brilliant III Ultra-Fast SYBR Green QPCR Master Mix (Agilent Technologies, Mississauga, ON, Canada). The sequence-specific primers for *ABCC2* (the gene encoding human MRP2) were 5’- AGAGCTGGC CCTTGTACTCA -3’ and 5’-AGGGACAGGAACCAGGAGTT - 3’. Gene expression was normalized to glyceraldehyde 3-phosphate dehydrogenase (*GAPDH*) gene expression as previously reported [32,33].

### Drug cytotoxicity assays

Caco-2 cells were seeded in 96-well plates at a density of 7,500 cells/well. Cells were grown for 24 h, after which etoposide, cisplatin or doxorubicin was added to the appropriate wells in varying concentrations (from 10 to 500 μM) and cells incubated for 84 h. For experiments with nucleotide stimulations, 100 μM ATP was added to the appropriate wells 6 h prior to the addition of the cytotoxic drug. Fresh nucleotides were added every 24h. Resistance to the different drugs was determined by MTT cell viability assay as described by the manufacturer. Briefly, 20 μl of 10 mg/ml of MTT were added to each well and incubated at 37°C for 4 h. The media was removed, 100 μl of DMSO was added to each well and the plates were agitated on a shaker for 5 min at 1,500 rpm in order to solubilize formazan. Optical densities were measured using a microplate reader (Molecular Devices VERSAmax microplate reader, Guelph, ON, Canada) at 560 nm and at 670 nm to determine background signal.

### Western blotting

Caco-2 cells were stimulated with 100 μM ATP for 6 and 18 h. Cells were washed with ice-cold PBS and lysed in Triton buffer (40 mM Tris (pH 7.5), 150 mM NaCl, 1 mM EDTA, 1% Triton X-100, 0.2 mM sodium orthovanadate, 40 mM glycerophosphate, 0.1 mM phenylmethanesulfonyl fluoride and protease inhibitor mixture from Sigma-Aldrich). Protein concentration was determined using the Bio-Rad Protein Assay reagent. Samples were heated for 5 min at 95°C, subjected to 7% SDS-PAGE and transferred onto polyvinylidene fluoride membranes for protein immunoblotting as previously described [32,33]. Immunoblotting for MRP2 was performed using a 1/1,000 dilution of rabbit polyclonal anti-MRP2 and the specific protein band was detected using a 1:10,000 dilution of HRP-conjugated donkey anti-rabbit IgG and visualized on autoradiographic film using the Millipore ECL chemiluminescence system. Signal was normalized as described with 1/5,000 dilution of rabbit anti-β-tubulin antibody [32,33].

### Measurement of ecto-nucleotidase activity

The ATPase activity was determined in adherent Caco-2 cells grown in 24-wells plate following the protocol described by Wink et al. [34]. Briefly, adherent Caco-2 cells were incubated the reaction medium (80 mM Tris-base, 5 mM CaCl2, 150 mM NaCl (pH 7.4)), and enzymatic the enzymatic reaction initiated by the addition of 0.2 mM ATP for 20 min at 37°C. The release of inorganic phosphate (P_i_) in the incubation medium was measured by the Malachite Green method [35], and the protein concentration of cell homogenate was determined as described above. The specific activity was expressed as nmol P_i_ released/min/mg protein.

### Statistical analysis

Results are expressed as the mean ± standard error of the mean (SEM). Statistical significance was determined using an unpaired *t* test or analysis of variance (ANOVA) with Dunnett's multiple comparison post-test as described in figure legends. The number of replicates for each experiment is also presented in figure legends. IC_50_ values were extrapolated from survival curves using non-linear regression analysis from the mean of three to four experiments. The relative resistance factor (RR) was determined by dividing the IC_50_ of stimulated or shMRP2-transfected cells by the IC_50_ of control cells, as previously reported [36,37].

## Results and Discussion

### Upregulation of MRP2 expression by ATP is mediated at the transcriptional and protein level by P2Y receptors

**Fig 1:**
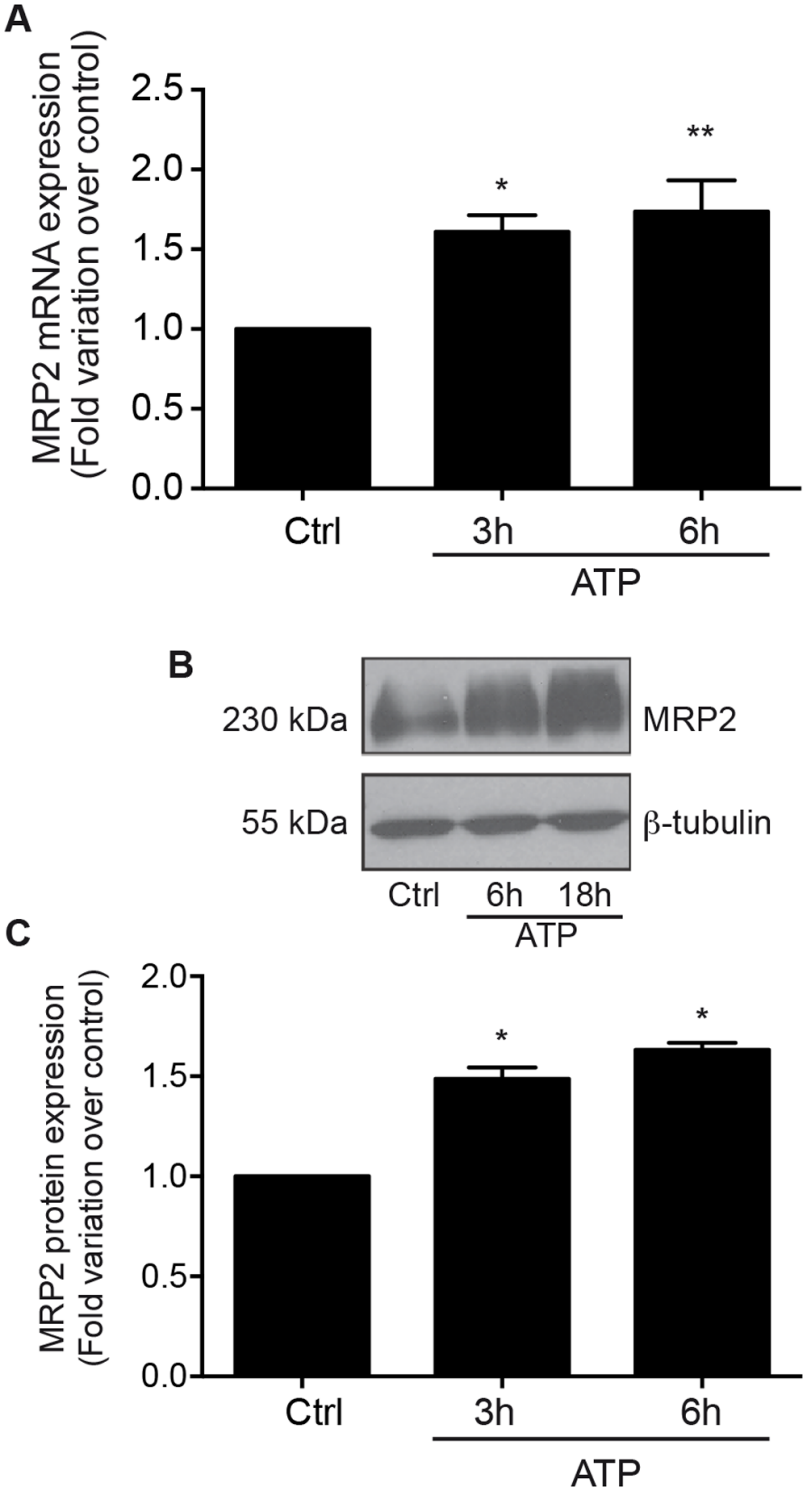
Extracellular ATP upregulates the expression of MRP2 in enterocyte-like Caco-2 cells. Human intestinal adenocarcinoma Caco-2 cells were stimulated with 100 μM ATP for 3 or 6 h, or with control vehicle only (Ctrl). **(A)** Stimulation with ATP significantly increased *ABCC2* gene expression (encodes for MRP2) by 1.5 to 2-fold compared to non-stimulated Ctrl as determined by qRT-PCR analysis. Results are presented as the mean ± SEM of five individual experiments performed in duplicate. Statistical significance was determined by one-way ANOVA with Dunnett's multiple comparison post-test. * p < 0.05, ** p < 0.01 as compared with Ctrl. **(B)** Typical Western blot result is showing enhanced MRP2 expression in ATP-stimulated cells. **(C)** Densitometric analysis revealed that 100 μM ATP induced MRP2 protein expression by more than 1.5-fold after 6 and 18 h of stimulation compared with control (Ctrl). Results are presented as the mean ± SEM of five separate experiments. Statistical significance was determined by one-way ANOVA with Dunnett's multiple comparison post-test. * p < 0.05 as compared with Ctrl.

**Fig 2:**
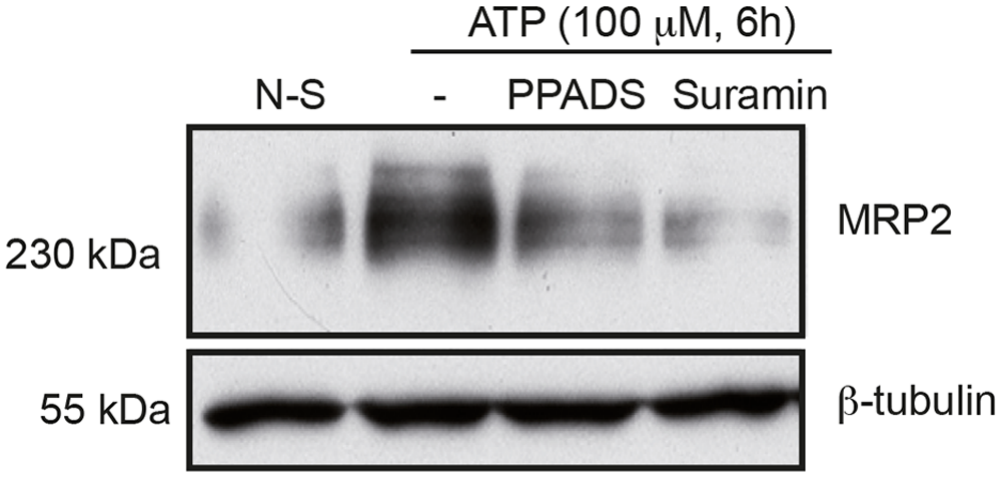
ATP-dependent stimulation of MRP2 protein expression is strongly reduced in the presence of the P2 receptor antagonist Suramin. Caco-2 cells were treated with 100 μM PPADS or Suramin 30 min prior to the addition of 100 μM ATP for 6 h. MRP2 expression was analyzed by Western blotting. ATP stimulated the expression of MRP2 as compared to non-stimulated (N-S) cells, whereas the addition of Suramin prior to the ATP stimulation strongly decreased MRP2 expression compared to ATP-stimulated cells in the presence of vehicle (DMSO (-)) only. The presented blot is typical of three separate sets of experiments.

The solid tumor microenvironment is rich in growth factors, cytokines, and chemokines. These factors contribute to the formation of an inflammatory microenvironment that stimulate tumorigenesis [1,38]. Extracellular ATP is also found in abundance in the tumor vicinity [6,7] where it can promote the proliferation of lung, breast and colon cancer cells [13,39,40] as well as supporting the invasion of prostate cancer cells and migration of lung and cancerous IECs [41–43]. In this study, we proposed that the presence of ATP in the tumor vicinity could contribute to drug resistance as often reported in patients under chemotherapy treatments for colorectal cancer [44]. In fact, analysis of MRP2 mRNA expression in IECs stimulated with 100 μM ATP for 3 and 6 h using qRT-PCR revealed that nucleotide treatments led to a 1.5- to 2-fold increase in the expression of MRP2 transcript (Fig 1A). Given that MRP2 is regulated at the transcriptional level, MRP2 protein expression was subsequently analyzed. Stimulation of Caco-2 cells with 100 μM ATP for 6 or 18 h increased MRP2 protein expression, as determined by immunoblotting (Fig 1B and 1C). The expression of MRP2 was also upregulated by 1.5- to 2-fold following nucleotide treatment, as assessed by densitometry (Fig 1C). To validate that the upregulation of MRP2 expression in IECs is indeed regulated by P2 nucleotide receptors, Caco-2 cells were treated with two known general antagonists of P2 receptors, namely PPADS and suramin. Following the addition of the latter to Caco-2 cells prior to stimulation by 100 μM ATP for 6h (Fig 2), Western blot analysis revealed that PPADS had no significant effect on MRP2 protein expression whereas the presence of suramin led to a marked reduction in ATP-dependent induction of MRP2 expression (Fig 2). Considering that ATP is the primary agonist of human P2X and P2Y_2 and 11_ receptors, and that suramin is a more potent antagonist of P2Y_2_ while PPADS is a more potent antagonist of P2Y_1, 4, 6_ and P2Y_13_ as well as P2X1, 2, 3 and 5 receptors [45,46], the present result suggests that P2Y_2_ may be involved in the regulation of MRP2 expression. Although similar results were obtained with other cancerous intestinal epithelial cell lines T84, DLD-1, HT-29 and HCT116 (data not shown), our focus was placed on Caco-2 cells since these cells display typical features of enterocytes [47]. Such increased expression in ATP-sensitive P2 receptors has previously been reported in both human colorectal carcinoma cells and cell lines [48]. Indeed, the expression of the P2Y_2_ receptor, as well as P2Y_4_, was reported to be upregulated in human colon cancer tissues [49]. Activation of these receptors has been associated with the regulation of cancerous cell growth and resistance to apoptosis [18]. Similarly to P2Y_2_ receptor expression, cancerous tissues isolated from CRC patients showed increased expression of MRP2 transcript levels as compared to non-cancerous margins [29]. Although ATP appears to be the main soluble factor contributing to the upregulation of MRP2 expression in Caco-2 cells, we cannot ruled out that adenosine 5’-diphosphate (ADP) could play a minor role in this process. Because ADP could be generated through the hydrolysis of ATP by ecto-nucleoside triphosphate diphosphohydrolase (E-NTPDase, EC 3.6.1.5) present at the surface of Caco-2 cells [33,50], we determined that adherent Caco-2 cells have an ATPase activity of 1.32 ± 0.04 nmol/min/mg protein. The ATPase activity is the average ± SEM of three experiments done in triplicate. Our findings further suggest that stimulation of cancerous IECs with ATP increases the expression of MRP2, a protein known for its role in cell resistance to a number of chemotherapeutic agents used in the treatment of colorectal cancer [26].

### Modulation of MRP2 expression is regulated by the MEK/ERK signaling cascade

**Fig 3:**
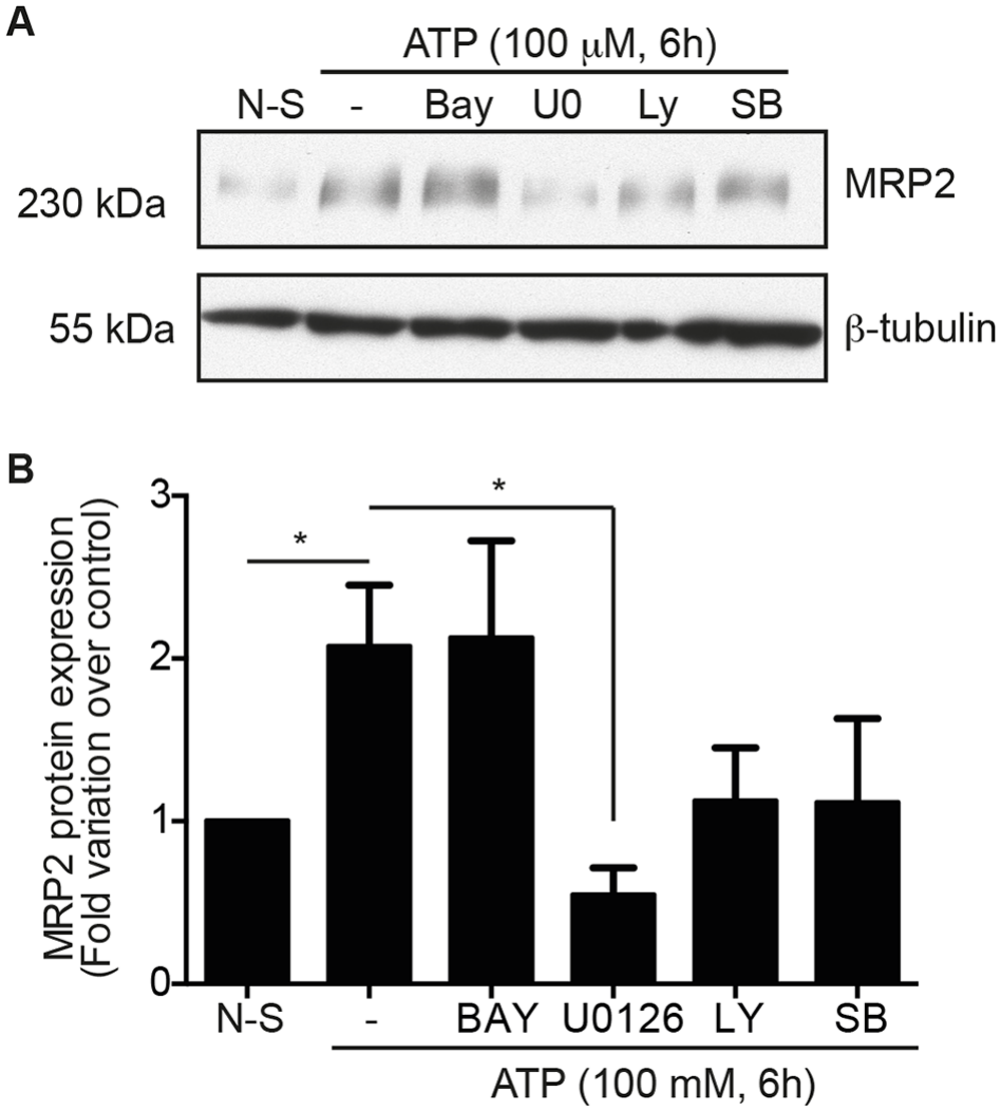
MRP2 expression is regulated by MEK/ERK signaling cascade. Caco-2 cells were pretreated with NFκB (2 μM, Bay, BAY11-7082), MEK1/2 (10 μM, U0, U0126), PI3K (20 μM, LY, LY294003) and p38 (20 μM, SB, SB203580) inhibitors for 30 minutes and stimulated with 100 μM ATP for 6 h. **(A)** A typical Western blot against MRP2 is displayed from which **(B)** densitometry analysis showed a significant reduction in MRP2 expression in the presence of U0126, a selective MEK 1/2 inhibitor. Cells pretreated with U0126 led to a 75% reduction in the expression of MRP2 compared to ATP-stimulated cells only (-). Results are presented as the mean ± SEM of three separate experiments performed in duplicate. Statistical significance was determined by an unpaired *t*-test, where * p < 0.05 vs. non-stimulated (N-S) or ATP-stimulated cells as indicated on figure.

It is well documented that extracellular ATP, through the activation of P2Y receptors, stimulates numerous intracellular signaling pathways [13,30,41,51]. These pathways are predominantly associated with ATP-dependent regulation of proliferation [13,14], cell motility and microtubule reorganization [41], secretion of inflammatory factors such as PGE_2_ in an NFκB-dependent manner [30] and modulation of oxalate, electrolytes and glucose transport [52–55]. To determine the signaling cascades involved in the regulation of MRP2 expression, Caco-2 cells were pretreated with various inhibitors, notably BAY11-7082 (NFκB), U0126 (MEK 1/2), LY294002 (PI3K) and SB203580 (p38) for 30 minutes and then stimulated with 100 μM ATP for 6 hours. Cells treated with the MEK/ERK signaling cascade inhibitor U0126 exhibited a significant decrease in MRP2 expression when compared to ATP-stimulated cells (Fig 3A). Densitometry analysis confirmed that inhibition of the MEK-ERK pathway significantly reduced MRP2 expression by 2-fold (Fig 3B). Of note, inhibition of the ATP-dependent activation of the ERK1/2 pathway has previously been associated with a reduction in colon adenocarcinoma cell proliferation [13] and, conversely, to increased viability of human endometrial stromal cells [56]. The ERK pathway has also been associated with resistance to cancer treatment by regulating the expression of multidrug resistant proteins (MDR), including MRP2, in pancreatic cancer cells [57] as well as in Caco-2 cells [58]. Our results showed that ERK signaling is involved in ATP-induced MRP2 expression in Caco-2 human adenocarcinoma cells.

### The increased expression of MRP2 in cancer cells provides resistance to the cytotoxic drug etoposide

**Fig 4:**
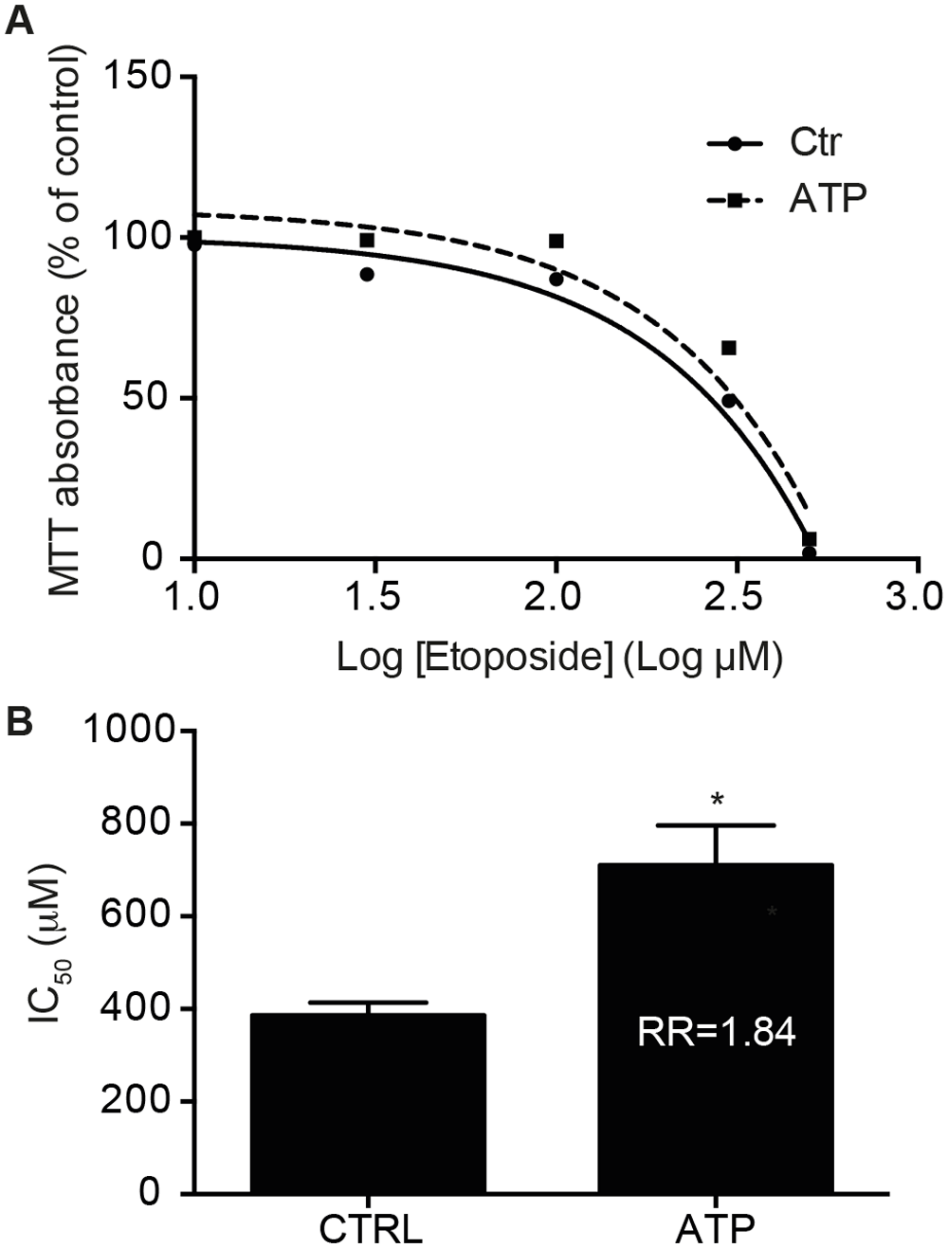
Stimulation of Caco-2 cells with ATP increases resistance to the anti-cancer drug etoposide. Caco-2 cells were incubated with increasing concentrations of etoposide for 84 h in the presence or absence of 100 μM ATP added every 24 h. MTT cell viability assay was used to determine sensitivity to the drug. **(A)** A dose-response curve was fitted to the data to determine the toxicity (IC_50_ value) of the drug. Results are presented as a non-linear survival curve of a typical response. **(B)** IC_50_ and RR values are presented on the histogram and results represent the means ± SEM of three to four independent experiments performed in triplicate. Statistical significance was determined by unpaired *t*-test, where * p < 0.05 as compared with Ctrl.

Increased expression of MRP2 in colon cancer has been associated with resistance to cisplatin, but not to 5-FU [29], even though *ATP-binding cassette sub-family C member 2* (*ABCC2*) haplotype was shown as a predictor of variability in the pharmacokinetic response to FOLFIRI regimen in Japanese CRC patients, which includes 5-FU [59]. Considering that MRP2 can export a variety of compounds, notably cytotoxic drugs [60–62], we studied the effect of ATP on cell survival of Caco-2 cells upon treatment with the anti-cancer drug etoposide. In a first instance, the resistance of Caco-2 cells to various concentrations of this drug was measured with or without ATP stimulation. Cell survival was determined by the MTT colorimetric test. An increased cell survival was observed in cells stimulated with ATP compared to non-stimulated cells (Fig 4A), which translated into a significant increase in IC_50_ values (Fig 4B). The relative resistance (RR) values of the various conditions were also calculated and were found to be higher in cells stimulated with ATP compared to non-stimulated cells. The RR value of 1.84 indicates that the ATP-stimulated cells were 1.84 times more resistant to the anti-cancer drug etoposide (Fig 4B). This finding suggest that the ATP-dependent stimulation of MRP2 expression led to an increased resistance of Caco-2 human colorectal adenocarcinoma cells to etoposide, and thus to possible involvement of extracellular ATP and P2Y receptors in CRC anti-cancer drug therapy resistance. Taking into account that MRP2 expression was previously associated with resistance to cisplatin, these results are thus concordant with the hypothesis that MRP2 exports chemotherapeutic drugs out of cancer cells [27,28,61] and thus favors their survival. Hence, these findings are also in accordance with the proposed role for P2Y receptors in tumor development through resistance to treatment as previously reported [13].

### Invalidation of MRP2 expression leads to a decreased resistance of cells to chemotherapeutic drugs

**Fig 5:**
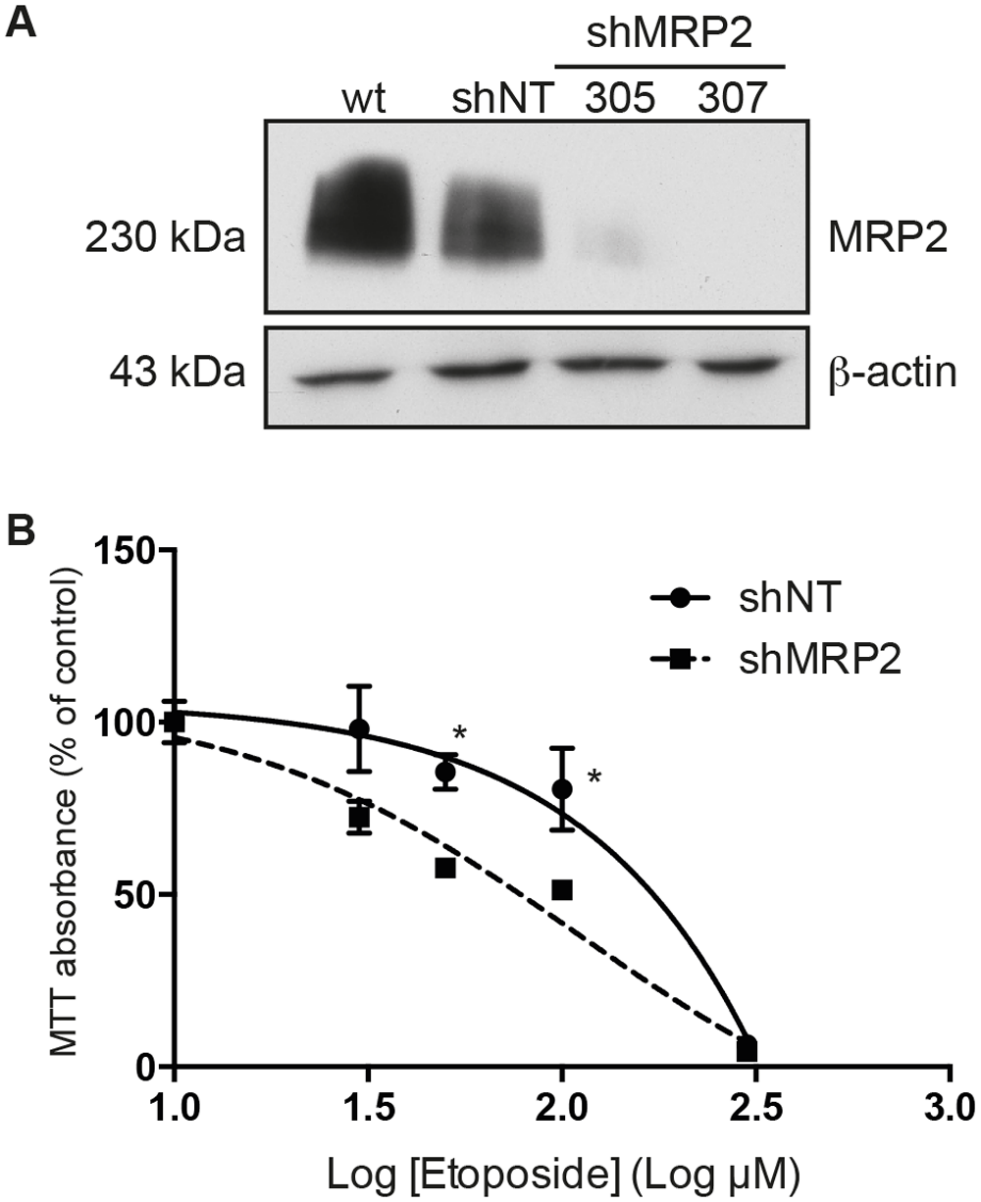
The down-regulation of MRP2 expression by shRNA renders Caco-2 cells more sensitive to etoposide. **(A)** Western blot analysis was used to assess the down-regulation of MRP2 protein expression in the presence of two shRNAs directed against the protein. Down-regulation was achieved by lentiviral infection of Caco-2 cells as previously described [31]. shRNA directed against MRP2 (sh305 and sh307) abolished protein expression by 90–100% comparatively to cells expressing a non-targeting shRNA (shNT). **(B)** Caco-2 cells stably expressing shNT or shMRP2 (#305) were incubated with the cytotoxic drug etoposide for 84 h. Sensitivity to the anti-cancer drug was determined by the MTT cell viability assay. A dose-response curve was fitted to the data to determine the toxicity (IC_50_ value) of the drugs. The non-linear survival curves are presented as the mean ± SEM of four experiments performed in triplicate. Statistical significance was calculated using multiple *t*-test comparisons, where * p < 0.05 as compared with shNT. Inhibition of human shMRP2 expression reduced the resistance of Caco-2 cells to etoposide compared to control cells. IC_50_ and RR values are presented in Table 1.

Following the observation that ATP-stimulated Caco-2 cells were exhibiting an increase in MRP2 expression and were more resistant to etoposide cytotoxicity, we investigated whether the inhibition of MRP2 could have the opposite effect. To verify this hypothesis, MRP2 expression was invalidated in Caco-2 cells using shRNA. Lentiviral infection of Caco-2 cells with shRNA directed against MRP2 (sh305 and sh307) abolished protein expression by 90–100% (Fig 5A). As shown in Fig 5B, Caco-2 cells invalidated for MRP2 were less resistant to etoposide treatment, with IC_50_ values of 328.2 μM and 108.5 μM for shNT and shMRP2, respectively, for an RR value of 0.33 (Table 1). Inhibition of MRP2 expression also sensitized Caco-2 cells to the cytotoxic effect of cisplatin and doxorubicin (Table 1). In the presence of cisplatin or doxorubicin, Caco-2 cells invalidated for MRP2 expression were approximately 2.5 times less resistant to treatment as shown by respective RR values of 0.36 and 0.40 for cisplatin and doxorubicin, respectively (Table 1). These results are thus concordant with the hypothesis that MRP2 exports chemotherapeutic drugs out of colorectal cancer cells and thus favors their survival. An increased expression of this transporter by extracellular ATP further increases this resistance. On the other hand, the downregulation of MRP2 by shRNA leads to a decrease in the resistance of cancer cells to the drugs and subsequently decreases their cell survival.

**Table 1:** IC_50_ and relative resistance (RR) values measured in response to etoposide, cisplatin and doxorubicin treatment of Caco-2 cells stably expressing non-target shRNA (shNT) or shMRP2.

	shNT	shMRP2	
	IC_50_ (μM)	IC_50_ (μM)	RR
Etoposide	328.2	108.5	0.33
Cisplatin	87.8	31.8	0.36
Doxorubicin	32.8	13.1	0.40

Caco-2 cells stably expressing shNT or shMRP2 (#305) were incubated with the cytotoxic drugs for 84 h. Sensitivity to the anti-cancer drug was determined by the MTT cell viability assay. Dose-response curves were fitted to the data to determine the toxicity (IC_50_ value) of the drugs. Inhibition of human shMRP2 expression reduced the resistance of Caco-2 cells to etoposide, cisplatin and doxorubicin compared to control cells.

## Conclusions

In the present study, we show a correlation between the stimulation of human colon adenocarcinoma cells with ATP through P2Y receptors and the activation of the MEK/ERK pathway as well as the expression of MRP2. In particular, we demonstrate that the activation of P2Y receptors by extracellular ATP induces the upregulation of MRP2 expression. This increased expression of MRP2 leads to the increased resistance and survival of intestinal cancerous Caco-2 cells to certain chemotherapeutic drugs used for the treatment of colorectal cancer. Although a role for P2Y receptors in tumor development through resistance to treatment has previously been proposed [13], this study is the first to propose a mechanism by which such an effect could be mediated. Hence, these findings call for further study to delineate the role of P2 receptors in cancer drug therapy and to develop novel therapies aimed at regulating P2 receptor activity. Given the heterogeneity of colorectal adenocarcinoma responses to anti-cancer drugs, screening of patients for P2 receptors and/or identification of mutant form of P2 receptors as well as for MRP2 expression could be beneficial in optimizing anti-cancer treatments.
